# Synergistic Microbicidal Effect of AUR and PEITC Against *Staphylococcus aureus* Skin Infection

**DOI:** 10.3389/fcimb.2022.927289

**Published:** 2022-06-14

**Authors:** Haoran Chen, Ning Yang, Liang Yu, Jiajia Li, Hui Zhang, Yahong Zheng, Mengran Xu, Yanyan Liu, Yi Yang, Jiabin Li

**Affiliations:** ^1^ Department of Infectious Disease, The First Affiliated Hospital of Anhui Medical University, Hefei, China; ^2^ Anhui Center for Surveillance of Bacterial Resistance, Hefei, China; ^3^ Institute of Bacterial Resistance, Anhui Medical University, Hefei, China; ^4^ The Center for Scientific Research, The First Affiliated Hospital of Anhui Medical University, Hefei, China

**Keywords:** auranofin (AUR), phenethyl isothiocyanate (PEITC), *Staphylococcus aureus*, thioredoxin reductase (TrxR), reactive oxygen species (ROS), skin infections

## Abstract

Given the increasing prevalence of *Staphylococcus aureus* antibiotic resistance, there is an urgent need to repurpose approved drugs with known pharmacology and toxicology as an alternative therapeutic strategy. We have reported that the sustained monotherapy of auranofin (AUR) inevitably resulted in reduced susceptibility or even the emergence of resistance to AUR in *S. aureus.* However, whether drug combination could increase antibacterial activity while preventing AUR resistance is still unknown. Here, we focused on the important role of AUR combined with phenethyl isothiocyanate (PEITC) in skin infection and determined the synergistic antimicrobial effect on *S. aureus* by using checkerboard assays and time-kill kinetics analysis. This synergistic antimicrobial activity correlated with increased reactive oxygen species (ROS) generation, disruption of bacterial cell structure, and inhibition of biofilm formation. We also showed that AUR synergized with PEITC effectively restored the susceptibility to AUR *via* regulating thioredoxin reductase (TrxR) and rescued mice from subcutaneous abscesses through eliminating *S. aureus* pathogens, including methicillin-resistant *S. aureus* (MRSA). Collectively, our study indicated that the AUR and PEITC combination had a synergistic antimicrobial impact on *S. aureus in vitro* and *in vivo*. These results suggest that AUR and PEITC treatment may be a promising option for *S. aureus* infection.

## Introduction

The skin is the largest and most exposed organ system of the human body, colonized by a diverse microbiota that is dominated by members of the *Staphylococcus*, *Corynebacterium*, *Streptococcus*, and *Propionibacterium* genera (Grice and Segre, 2011; Byrd et al., 2018; Parlet et al., 2019). In the *Staphylococcus* genus, *Staphylococcus aureus* is responsible for most acute and chronic infections, and it is often included on lists of the most problematic pathogens. *S. aureus* also contributes to the pathogenesis of skin-associated diseases, and may cause aggravation of atopic dermatitis (AD) and systemic lupus erythematosus (SLE) (Sirobhushanam et al., 2020; Cavalcante et al., 2021). As the use of antibiotics has increased, the spread of resistant strains of *S. aureus* has become a public health crisis. Methicillin-resistant *S. aureus* (MRSA) is one of the most prevalent antibiotic-resistant strains and accounts for 60% of clinically isolated *S. aureus* (Romaniuk and Cegelski, 2015). Clinical manifestation of MRSA infection ranges from skin and soft tissue infections (SSTIs) to severe invasive disease with high morbidity and mortality (Lee et al., 2018). Even treatment with antibiotics like vancomycin and daptomycin, which are considered last‐resort antibiotics for MRSA infections, have become ineffective (Bayer et al., 2013; McGuinness et al., 2017). Moreover, the bacterial biofilm formation is a serious clinical issue, that represents enormous levels of antibiotic resistance. In samples of patients with chronic, non-healing cutaneous wounds, over half were shown to harbor biofilms (Ward et al., 2015). Thus, there is a critical need for novel antimicrobial agents.

**Figure 1:**
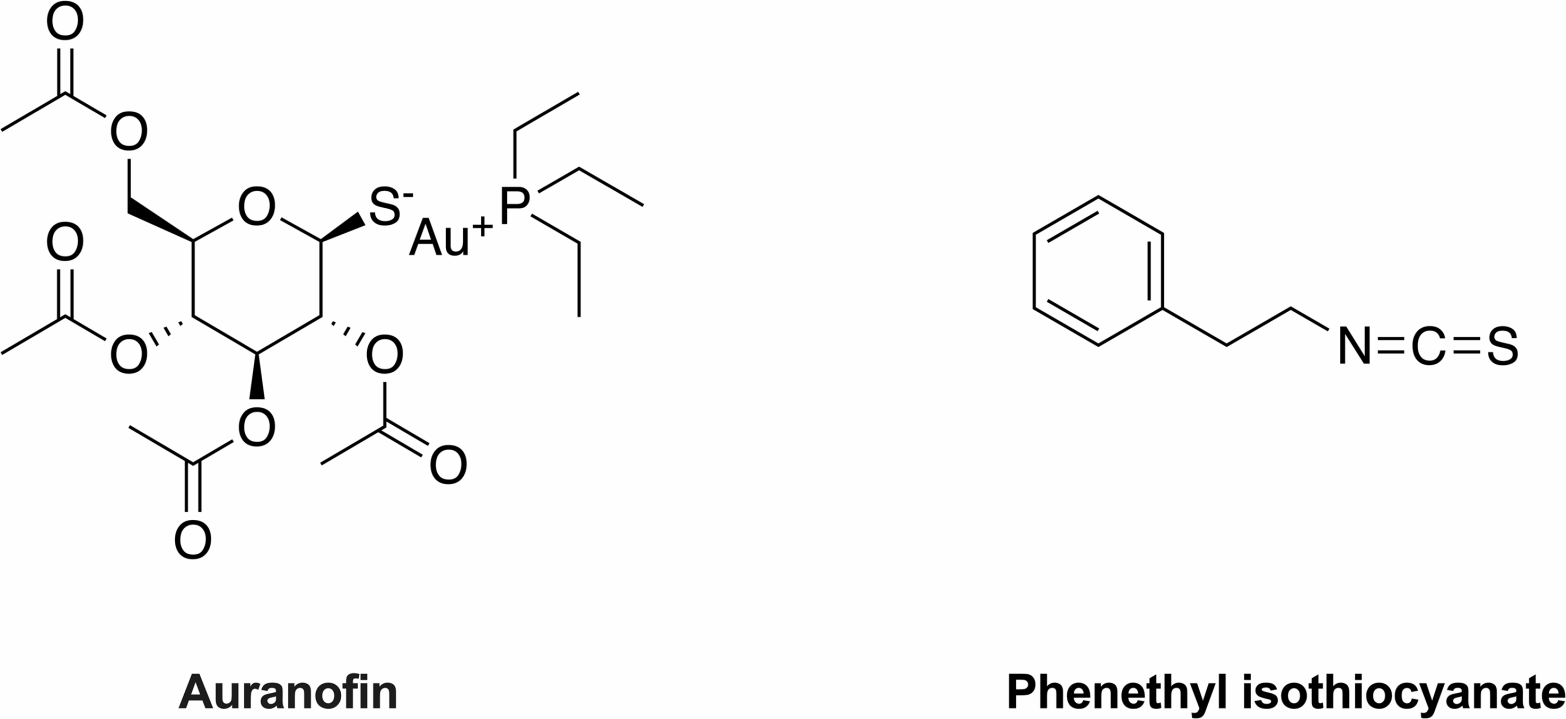
The structure of auranofin and phenethyl isothiocyanate.

Taking into account the discovery and development of new antimicrobial agents is characterized by high cost, long trial phases and low success rate, the repurposing of FDA-approved drugs is a very productive alternative method. Recently, metal compounds have been reported to have an inhibitory effect on SARS-CoV-2 replication, which exhibits the potential pharmacological value for this class of compounds (Yuan et al., 2020). Auranofin (AUR), a gold-containing compound with phosphine and thiol ligands in alinear arrangement (**Figure 1**), is prescribed for the treatment of rheumatoid arthritis in 1985 (Glennås et al., 1997). Thioredoxin reductase (TrxR), part of the antioxidant thioredoxin (Trx) system, is essential for *S. aureus* growth (Uziel et al., 2004; Harbut et al., 2015). Previous findings have shown that AUR exhibits significant antimicrobial activity against Gram-positive pathogens by inhibiting TrxR and disrupting Trx-TrxR function to induce reactive oxygen species (ROS) accumulation, then eventually causing bacterial death (Feng et al., 2021; Abutaleb and Seleem, 2020). However, our earlier studies demonstrated that sustained AUR monotherapy can induce TrxR gene (*trx*B) mutations at high dose, which probably lead to treatment resistance *via* reduction of the binding of metal cations to the active site cysteines of TrxR (Chen et al., 2020). Therefore, antimicrobial combination are promising ways to solve this problem by achieving the desired therapeutic effect at a lower concentration and preventing the emergence of resistance.

ROS are implicated in bactericidal antibiotics that induce bacterial cell death (Kohanski et al., 2007; Van Acker and Coenye, 2017). Current evidence suggests that all isothiocynates (ITCs) were found to cause oxidative stress and intracellular ROS generation (Pocasap et al., 2018). Phenethyl isothiocyanate (PEITC), one of the most promising ITCs, is formed by enzymatic hydrolysis of glucosinolates present in cruciferous vegetables (**Figure 1**) and has its unique biological features charactering as high bioavailability and can be rapidly absorbed (Lv et al., 2020a). Studies have found that oral administration of PEITC has chemopreventive and chemotherapeutic potential against multiple cancers in human clinical trials (Gupta et al., 2015; Lv et al., 2020b). PEITC is recently shown to effectively inhibit various Gram-positive bacteria, but the exact mechanism has not been clearly elucidated (Jang et al., 2010; Nowicki et al., 2019). Collectively, both AUR and PEITC induce ROS accumulation and upset the redox balance, although their mechanistic pathways are different. These findings suggest that ROS generation could be a potential mechanism for cellular death caused by the combined action of AUR and PEITC.

To identify new therapies against *S. aureus* skin infections and define the mechanistic basis for their effects, we hypothesized that PEITC may increase the antimicrobial activity of AUR and prevent AUR resistance. In the present study, we report that AUR combined with PEITC has a synergistic antimicrobial effect against *S. aureus* infections *in vitro* and *in vivo*. By combining ROS assay, scanning electron microscope (SEM), and antibiofilm assay, we found that hydroxyl radical formation, bacterial cell membrane integrity disruption, and biofilm inhibition are key factors involved. Notably, AUR synergized with PEITC enhanced clearance of *S. aureus* in infection sites and rescued antimicrobial activity of AUR to suppress the development of resistance. This novel drug combination should be considered for future clinical study of infections caused by antibiotic-resistant *S. aureus*.

## Materials and Methods

### Bacterial Isolates, Drugs, and Mice

*S. aureus* RN450, ATCC6538, methicillin-resistant *S. aureus* (MRSA) USA300, and vancomycin-resistant *S. aureus* (VRSA) Mu50 were wild-type standard strains (Novick, 1967; Garrido et al., 1982; Hiramatsu et al., 1997; Diep et al., 2006). The other three MRSA isolates, BN226, BN295, and BN508, were clinical wild-type pathogens. All clinical wild-type pathogens were isolated from the wound secretion of inpatients at the First Affiliated Hospital of Anhui Medical University. All isolates (from the Anhui Center for Surveillance of Bacterial Resistance-Huinet, Hefei, China) were grown in tryptic soy broth (TSB) broth medium (Solarbio, Shanghai, China) at 37°C. PEITC was obtained from Sigma-Aldrich (St. Louis, MO, USA). Auranofin was ordered from Yuanye Bio-Technology Co., Ltd. (Shanghai). Other reagents were obtained from Sigma-Aldrich. All prepared solutions were stored at -20°C within 1 month. Wild-type BALB/c mice were purchased from the Experimental Animal Center of Anhui Province (Hefei, China). All experiments involving mice were approved by the Institutional Animal Care and Use Committee of Anhui Medical University (Hefei, China).

### Susceptibility Testing

The minimum inhibitory concentrations (MICs) of AUR and PEITC were measured using the broth microdilution method, as recommended by the Clinical and Laboratory Standards Institute (CLSI) (Clinical and Laboratory Standards Institute [CLSI], 2021). Overnight cultures were diluted 200-fold into fresh cation adjusted Muller Hinton Broth (CAMHB; Sigma-Aldrich, USA), and grown to exponential phase OD_600_ ~0.3 (the optical density of growth cultures was measured at 600 nm) at ~5×10^8^ CFU/mL, after which the cultures were diluted in broth to a final bacterial inoculum of 5×10^5^ CFU/mL. Various amounts of the drugs were mixed and incubated at 37°C overnight. The MICs were defined as the lowest drug concentrations that inhibited visible bacteria growth.

### Checkerboard Assays

The synergistic effect was evaluated using the microdilution checkerboard assay. In brief, bacteria were cultured in CAMHB overnight at constant rotation (220 rpm) at 37°C. The bacterial density was adjusted to a ~5**×**10^5^ CFU/mL bacterial suspension and checked by CFU counting on agar plates. AUR, PEITC, or both drugs were added in triplicate to individual wells of a flat-bottomed 96-well plate and 2-fold serial dilution was performed, followed by the addition of prepared bacterial inoculum. The microwell plates were incubated at 37°C overnight before reading. Wells with no drugs served as growth controls and wells with medium only served as background controls.

The fractional inhibitory concentration index (FICI) was calculated as follows: FICI = FIC_A_ + FIC_B_ = (MIC of drug_A_ in combination/MIC of drug_A_ alone) + (MIC of drug_B_ in combination/MIC of drug_B_ alone). Synergy was defined when FICI ≤ 0.5, indifference was defined when FICI > 0.5 and < 4, and antagonism was defined when FICI ≥ 4 (Sun et al., 2020). All determinations were performed at least in triplicate on different days.

### Time-Kill Studies

Time-kill studies were conducted using AUR (1/2×MIC, 1×MIC, 2×MIC, and 4×MIC) in combination with PEITC (1× MIC), as previously described (Chen et al., 2021). In brief, bacterial strains were cultivated overnight and diluted 1:200 in fresh broth at 37°C on a shaker for 3 h to reach log phase. They were then prepared for MIC determination and treated with AUR, PEITC, or both. Samples and untreated cultures were removed at various times after 10–10^5^ fold dilutions with saline. The diluted samples were aseptically placed (10 µL) on agar plates incubated at 37°C overnight to measure the viable colony cell counts (CFU/mL). Controls (untreated) were set for each experiment. All assays were performed in triplicate on different days.

### RNA Extraction, Reverse Transcription, and Quantitative Real-Time Polymerase Chain Reaction (qRT-PCR) of Gene Expression

Pretreated cultures were incubated overnight and diluted 100-fold in fresh broth for the OD_600_ of ~0.3 (log-phase) on a shaker at 37°C. The cell wells of *S. aureus* were then lysed using lysozyme (20 mg/mL) and lysostaphin (200 U/mL) for 3 h. RNA was extracted using RNeasy kit (Qiagen, Hilden, Germany) by strictly following the manufacturer’s protocol. For reverse transcription, cDNA was synthesized using PrimeScript cDNA synthesis kit (TaKaRa, Kyoto, Japan). Trx and TrxR genes (*trx*A and *trx*B) were detected by a three-step real-time PCR system (Light Cycler 96; Roche, Basel, Switzerland). Primers for qRT-PCR are listed in **Table S1**. Fold changes in *trx*B and *trx*A expression levels were normalized to the 16S rRNA internal control and determined by the 2_–ΔΔCT_ calculation method. Reactions were run in triplicate.

### ROS Assay

The fluorescent probe, carboxy-H2DCFDA (Thermo Fisher, Waltham, MA, USA), was used to measure intracellular ROS accumulation. Bacterial fluorescence intensity was analyzed using a flow cytometer (Beckman Coulter CyAn ADP analyzer; Brea, CA, USA) (Bass et al., 1983; Chen et al., 2021). Exponentially growing cultures (RN450 and USA300) were treated with AUR (4×MIC) alone, PEITC (1×MIC) alone, or in combination for 4 h. Carboxy-H2DCFDA was added to the treated or untreated samples at a final concentration of 10 µM to detect intracellular ROS levels. Samples (200 µL) were washed twice with precooled 1× phosphate-buffered saline (PBS) to scavenge the agents and concentrated by centrifugation at 4600 xg for 2 min. A total of 100,000 cells were analyzed by flow cytometry.

The fluorescent reporter dye, 3’-(p-hydroxyphenyl) fluorescein (HPF; Thermo Fisher, USA), which was oxidized by hydroxyl radicals with high specificity, was used (Wang et al., 2014). A suitably diluted stock solution of the dye (final HPF concentration, 5 μM) was added to treated or untreated cultures. Fluorescence (excitation, 490; emission, 515 nm) was measured using a Tecan Spark Multimode Microplate Reader (Tecan, Mannedorf, Switzerland). Cultures treated with hydrogen peroxide (H_2_O_2_), which induces hydroxyl radicals, were used as a positive control.

We next determined whether ROS (peroxide)-mediated rapid killing is a key factor for the AUR-PEITC drug combination. Exogenous addition of an effective ROS scavenger, such as dimethyl sulfoxide (DMSO), mitigates ROS accumulation. Cultures were treated and placed on agar plates containing DMSO (5%, v/v), as in the previously described time-kill experiment. Growth kinetics was also studied in DMSO alone (Supplementary Data).

### Cellular Morphology Study

To visualize any effect that the AUR-PEITC drug combination had on the growth and morphology of *S. aureus*, each strain was treated in the presence of AUR, PEITC alone, or together, or in broth as a positive growth control for 8 h. In brief, the cell suspension was washed three times in 1 mL of PBS. The bacterial pellets were then resuspended in 0.5 mL PBS with 2.5% glutaraldehyde and fixed for 2 h at 37°C. The slides were washed twice by immersion in 1×PBS for 10 min and then dried by stepwise placement in graded ethanol solutions (10, 25, 40, 60, 75, and 90%) for approximately 10 min or until completely dried. The samples were observed with a scanning electron microscope (SEM).

### Crystal Violet Biofilm Assay

Biofilm inhibitory effect was measured using crystal violet as previously described (Xiao et al., 2021). Briefly, overnight cultures were standardized to exponential phase OD_600_ ~0.3, and then diluted (1:100) with fresh broth. Samples (200 μL) were loaded into individual wells of 96-well plates and incubated at 37°C for 24 h. The medium was discarded, and the wells were washed three times with PBS. Next, the bacterial biofilms were fixed with 95% formalin for 15 min and stained with 0.1% crystal violet solution for 20 min. Then, the excess stain was removed by washing twice with PBS, the stained biofilms were dried for 1 h and extracted with 33% glacial acetic acid. The amount of biofilm produced was quantified by measuring the optical density at 590 nm using a Tecan Spark Multimode Microplate Reader (Tecan, Mannedorf, Switzerland). The percentage of biofilm eradication and inhibition was determined according to the following equation:

The rate of biofilm eradication/inhibition (%) = (1 – OD_test_/OD_control_) × 100%

### Murine Skin Infection Model

Murine *S. aureus* skin infection models were generated as previously described to evaluate the effect of combined AUR and PEITC treatment on antibacterial activity *in vivo* (Xiao et al., 2021). The mice were anesthetized and the hair on the upper back of each mouse (approximately 2×2 cm^2^) was shaved off 24 h before modeling. Cultures were prepared by resuspending at a density of 1×10^10^ CFU/mL. Eight-week-old BALB/c mice (eight per group) were anesthetized with isoflurane and injected in the back subcutaneously with 50 μL of the bacterial cell suspension. After infection for 24 h, sterile 1× PBS, 1% AUR alone, 10 μM PEITC alone, or the AUR-PEITC combination was injected subcutaneously into the abscess site, and then the wounds were observed every two days (Mohammad et al., 2020; Osipitan et al., 2021).

After 2, 4, 6, 8, 10, and 12 days of therapy, the abscesses were imaged, and the weights and survival of mice were recorded to monitor the wound-healing process. The infected skin was assessed using Image J software. Mice were euthanized by CO_2_ asphyxiation at 8 days post-treatment. To determine bacterial density, samples of the abscess tissues were homogenized in 1 mL PBS and transferred into a sterile test tube for serial dilution. Viable counting was performed by culturing 100 μL tissue samples in broth agar and incubating them overnight at 37°C. A blank control (no treatment, PBS) group was set for each experiment. Statistical significance for bacterial killing in different treatment groups was calculated using one-way ANOVA and Tukey’s multiple comparisons (Tukey’s HSD).

### Statistical Analysis

Data represent the means from three independent experiments, and error bars represent standard errors of the means. All statistical analyses were performed using unpaired Student *t* tests for two groups and one- or two-way analyses of variance (ANOVAs) for multiple groups, with all data points showing a normal distribution. The results were considered statistically significant at P < 0.05. All graphs were generated using GraphPad Prism 8.0 (GraphPad Inc., San Diego, CA, USA), FlowJo version 10.4 (Ashland, OR, USA), and Adobe Illustrator CC 2018 (Adobe Systems Inc., USA).

## Results

### Antimicrobial Susceptibility of AUR and PEITC

AUR and PEITC were tested on various *S. aureus* strains, including those resistant to conventional antimicrobials such as methicillin and vancomycin. As shown in **Table 1**, the MIC values for all test strains were 0.125 or 0.25 μg/mL for AUR and 20 or 40 μg/mL for PEITC. AUR and PEITC MIC values were similar for all the antibiotic-sensitive *S. aureus*, MRSA, and VRSA pathogens. All strains were more sensitive to AUR than PEITC treatment.

**Table 1:** The antimicrobial susceptibility and combined effects of AUR and PEITC alone or in combination against *S. aureus* strains.

Bacterial isolate	Agent	MIC (μg/mL)		MIC_In combination_/MIC_singly_	FICI	Outcome
		Singly	In combination			
*S. aureus* RN450	AUR	0.25	0.03125	0.125	0.375	Synergy
	PEITC	20	5	0.25
*S. aureus* ATCC6538	AUR	0.125	0.015625	0.125	0.375	Synergy
	PEITC	40	10	0.25
MRSA USA300	AUR	0.125	0.015625	0.125	0.375	Synergy
	PEITC	40	10	0.25
MRSA BN226	AUR	0.25	0.03125	0.125	0.25	Synergy
	PEITC	40	5	0.125
MRSA BN295	AUR	0.25	0.015625	0.0625	0.3125	Synergy
	PEITC	20	5	0.25
MRSA BN508	AUR	0.125	0.03125	0.25	0.375	Synergy
	PEITC	40	5	0.125
VRSA Mu50	AUR	0.25	0.03125	0.125	0.375	Synergy
	PEITC	40	10	0.25

MIC, minimum inhibitory concentration; AUR, auranofin; PEITC, phenethyl isothiocyanate; FICI, fractional inhibitory concentration index. Data are from at least three independent experiments.

### The Synergistic Effect of AUR and PEITC Against *S. aureus* Isolates

**Figure 2:**
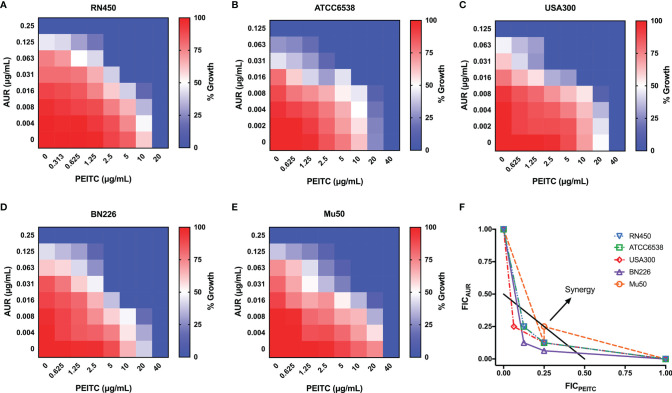
Synergistic effect between AUR and PEITC against *S. aureus* strains. Representative heat plots of microdilution checkerboard assays for the combination of AUR and PEITC against **(A)** *S. aureus* RN450, **(B)** *S. aureus* ATCC6538, **(C)** MRSA USA300, **(D)** MRSA BN226, and **(E)** VRSA Mu50. **(F)** Isobolograms of the combination of AUR and PEITC against different *S. aureus* strains. The black full line indicates ideal isobole, where drugs act additively and independently. Data points below this line indicate synergism. AUR, auranofin; PEITC, phenethyl isothiocyanate; FICI, fractional inhibitory concentration index. Data in **(A–E)** represent the mean OD_600_ of three biological replicates.

The synergistic effect of AUR in combination with PEITC was tested using checkerboard assays (**Table 1**). For antibiotic-sensitive *S. aureus*, the MIC value of AUR was reduced by 8-fold in the presence of (1/4×MIC) PEITC against *S. aureus* RN450 (**Figure 2A**), and the FICI was estimated to be 0.375, indicating that drug synergy had occurred (FICI ≤ 0.5, **Figure 2F**). Treatment of ATCC6538 gave the same results (**Figure 2B**). Importantly, PEITC sensitized the MRSA USA300 isolate to AUR by 8 fold, with dropping the PEITC MIC values from 40 μg/mL to 10 μg/mL (FICI= 0.375, **Figures 2C–F**), and the MRSA BN226 isolate to AUR by 8 fold, with dropping the MIC values of PEITC from 40 μg/mL to 5 μg/mL (FICI= 0.25, **Figures 2D–F**). Synergistic interactions were also observed against the MRSA clinical isolates, BN295 (FICI= 0.3125) and BN508 (FICI= 0.375) (**Supplementary Figure S1**). The combination of AUR and PEITC still showed typical synergism with a FICI of 0.375 for VRSA Mu50 (**Figures 2E, F**).

### PEITC Enhances AUR-Mediated Killing *In Vitro*

**Figure 3:**
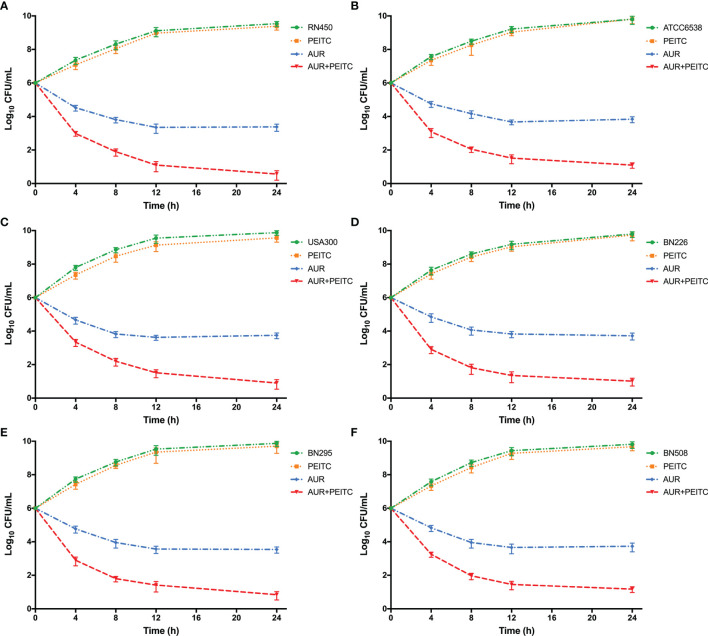
The synergistic killing of *S. aureus* by AUR in combination with PEITC. Time-kill curves for 4× MIC auranofin (AUR) or 1× MIC phenethyl isothiocyanate (PEITC) monotherapy or their combination therapy against *S. aureus* during 24 h incubation. **(A)** *S. aureus* RN450, **(B)** *S. aureus* ATCC6538, **(C)** MRSA USA300, **(D)** MRSA BN226, and **(E)** MRSA BN295, and **(F)** MRSA BN508. Data are from at least three independent experiments; error bars indicate the standard errors of the means.

We next examined whether PEITC could enhance the antimicrobial activity of AUR against *S. aureus* strains by using time-kill assays. PEITC in combination with various concentrations of AUR was used to treat *S. aureus* RN450 (**Figure S2A**). In the presence of 20 μg/mL PEITC, 4×, 2×, 1×, and 1/2×MIC AUR showed strong, moderate, low, and almost no killing of *S. aureus*, respectively. Thus, 4-fold MIC AUR was used to test the synergistic killing against other *S. aureus* pathogens. AUR (4×MIC) and PEITC combined treatment resulted in strong ~3.1 log_10_ CFU/mL killing of *S. aureus* RN450 (**Figure 3A**), ~2.8 log_10_ CFU/mL killing of *S. aureus* ATCC6538 (**Figure 3B**), and ~2.9 log_10_ CFU/mL killing of MRSA USA300 (**Figure 3C**) within 24 h. AUR-mediated lethality was also significantly enhanced when used with PEITC, and bacterial counts were at least 2.6 log_10_ CFU/mL lower for the three clinical MRSA pathogens than after AUR monotherapy **(Figures 3D–F**). Similar synergistic killing was observed for VRSA Mu50 (**Figure S2B**). PEITC alone had no effect on bacterial growth (**Figure 3**).

### PEITC Restores Susceptibility to AUR *via* TrxR

To assess the reversal of resistance to AUR by PEITC, AUR susceptibility studies were conducted using AUR (8×MIC) alone or with PEITC (20 μg/mL). Fold changes to the MICs of AUR after 96 h coincubation are shown in **Table 2**. Addition of PEITC restored the susceptibility of all tested strains to AUR (within 2-fold of initial MICs) at 96 h. Similar results were also observed in the untreated group. However, AUR alone resulted in a 16- to 64-fold increase in the MICs.

**Table 2:** Treatment of AUR with or without PEITC impact on minimum inhibitory concentrations at 96 h.

Bacterial isolate	MICs of AUR relative to their initial values (μg/mL)		
	Control^a^	AUR	AUR + PEITC
*S. aureus* RN450	1× MIC (0.25)	64× MIC (16)	1× MIC (0.25)
*S. aureus* ATCC6538	1× MIC (0.125)	64× MIC (8)	1× MIC (0.125)
MRSA USA300	1× MIC (0.125)	32× MIC (4)	1× MIC (0.125)
MRSA BN226	1× MIC (0.25)	32× MIC (8)	1× MIC (0.25)
MRSA BN295	1× MIC (0.25)	16× MIC (4)	1× MIC (0.25)
MRSA BN508	1× MIC (0.125)	64× MIC (8)	2× MIC (0.25)
VRSA Mu50	1× MIC (0.25)	16× MIC (4)	1× MIC (0.25)

The values of AUR MICs are shown in brackets. ^a^Controls, no treatment. AUR, auranofin; PEITC, phenethyl isothiocyanate. Data are from at least three independent experiments.

**Figure 4:**
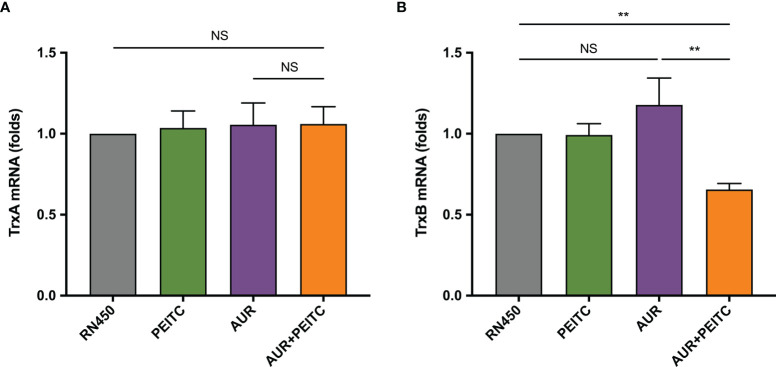
Fold changes in *trx*B expression in the combination of AUR and PEITC. Expression of **(A)** *trx*A and **(B)** *trx*B genes in *S. aureus* RN450. RN450 were untreated or treated with 8× MIC auranofin (AUR), 1× MIC phenethyl isothiocyanate (PEITC), or in combination for 96 h. **P < 0.01; NS, no significance. Data are from at least three independent experiments; error bars indicate standard errors of the means.

As previously mentioned, TrxR, the *S. aureus* target for AUR, is importance in maintaining systemic redox homeostasis. Thus, qRT-PCR was used to analyze expression of genes (*trx*A and *trx*B) encoding the antioxidant thioredoxin (Trx-TrxR) system for untreated or treated *S. aureus* RN450 as described above (**Figure 4**). By 96 h incubation of AUR monotherapy with high doses, the suppressing effect of AUR on *trx*B disappeared (**Figure 4B**). Not only that, the slight increase was observed in *trx*B gene expression, but this was not statistically significant (P > 0.05). In contrast, expression of *trx*B was significantly down-regulation after AUR-PEITC combination treatment (P < 0.01). However, *trx*A expression levels were no significant difference (P > 0.05) between AUR with and without PEITC (**Figure 4A**). Likewise, no alterations with PEITC monotherapy. On the basis of these data, we concluded that PEITC plays an important role in blocking AUR resistance.

### AUR-PEITC Combination Stimulates ROS Production

**Figure 5:**
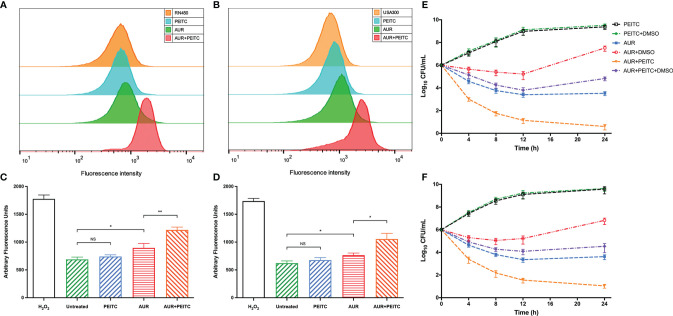
Role of ROS in mediating AUR combined with PEITC induced lethality of *S. aureus*. **(A)** *S. aureus* RN450 and **(B)** MRSA USA300 were incubated with 10 mM carboxy-H2DCFDA for 10 min before observation. Controls, no treatment at 4 h. **(C)** *S. aureus* RN450 and **(D)** MRSA USA300 were untreated or treated with 0.15% H_2_O_2_, 4× MIC auranofin (AUR), 1× MIC phenethyl isothiocyanate (PEITC), or in combination for 4 h. 3’-(p-hydroxyphenyl) fluorescein (HPF) was added after treatment, and the fluorescence was measured (490/515 nm). *P < 0.05; **P < 0.01; NS, no significance. **(E, F)** DMSO (5%) was added to diminish ROS levels. Combination-induced killing was decreased in the presence of DMSO for **(E)** *S. aureus* RN450 (P< 0.01) and **(F)** MRSA USA300 (P< 0.01) compared with no DMSO at 24 h. All panels show representative results from at least three independent experiments; error bars represent standard errors of the means.

Since both AUR and PEITC can influence the redox balance in bacteria, we explored whether PEITC increases AUR-stimulated ROS accumulation. Intracellular ROS levels were measured with carboxy-H2DCFDA in *S. aureus* RN450 and MRSA USA300. AUR alone had a weak effect on fluorescence levels, but comparative analysis showed that AUR in combination with PEITC significantly increased ROS accumulation for RN450 (**Figure 5A**) and UAS300 (**Figure 5B**). HFP was used to verify the combined effect of AUR and PEITC on hydroxyl radical formation. Similarly, significantly increases were observed in ROS levels for RN450 (**Figure 5C**) and USA300 (**Figure 5D**).

We next examined combined AUR and PEITC treatment for its effect on ROS-mediated programmed cell death. As with the time-killing results, synergistic killing was observed following combined PEITC and AUR treatment of *S. aureus* RN450 (**Figure 5E**) and MRSA USA300 (**Figure 5F**). However, adding 5% DMSO sharply diminished this combination-mediated synergistic killing against RN450 (increased by 4.1 log_10_ CFU/mL killing; P < 0.01) (**Figure 5E**), and against USA300 (increased by 3.6 log_10_ CFU/mL killing; P < 0.01) (**Figure 5F**). At this concentration, DMSO alone did not affect bacterial growth within 24 h (P > 0.05; **Figure S3**). Taken together, these data indicate that AUR and PEITC treatment causes strong killing by increasing intracellular ROS levels.

### Combined AUR and PEITC Treatment Exacerbates Morphological Damage

**Figure 6:**
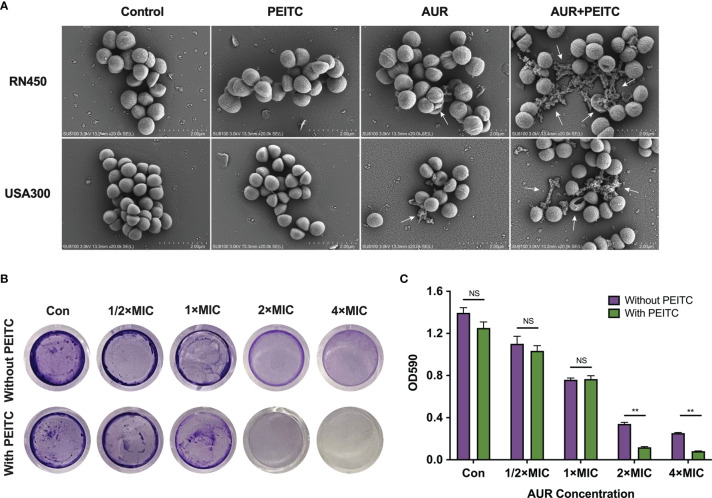
Impact of AUR and PEITC on bacterial morphology and biofilm formation. **(A)** Scanning electron micrographs (SEM) images of *S. aureus* RN450 and MRSA USA300 after treatment with (4× MIC) AUR alone, (1× MIC) PEITC alone or the AUR-PEITC combination. White arrows show morphological damage in bacteria. Scale bar: 2 μm. Controls, no treatment; AUR, auranofin; PEITC, phenethyl isothiocyanate. **(B)** Crystal violet staining of RN450 biofilms under different treatments. Biomass quantification of biofilms in **(C)** by measurement of absorbance at 590 nm. Error bars represent standard errors of the means. **P < 0.01; NS, no significance.

SEM experiments were performed to study morphological changes of *S. aureus* RN450, MRSA USA300, and VRSA Mu50 following treatment with AUR, PEITC, or both. Images showed that the RN450 and USA300 cell walls were intact in the absence of treatment and in response to PEITC monotherapy. AUR monotherapy caused some small ruptures on the cell walls. However, RN450 and USA300 treated with the AUR and PEITC combination both showed large-scale membrane disruptions with cratering, some cell lysis, extruded cell debris, and clumped structures (**Figure 6A**). The Mu50 cell surface was also extensively damaged and showed cell lysis in response to combination treatment (**Figure S4**).

### AUR Synergized With PEITC Against Biofilms Formation

We tested efficacy of the AUR and PEITC combination in eradicating established biofilms using *S. aureus* RN450 as a model. AUR monotherapy exhibited moderate biofilm inhibitory effects against RN450 in a dose-dependent manner, 21.1% of the biofilm was eradicated at 1/2×MIC AUR, 45.6% of the biofilm was eradicated at 1×MIC AUR, 75.6% of the biofilm was eradicated at 2×MIC AUR and 81.8% of the biofilm was eradicated at 4×MIC AUR compared to controls, respectively (**Figures 6B, C**). Obviously, the best inhibitory effect was observed when PEITC combined with AUR. The biofilm inhibition rates were 91.5% for PEITC combined with 2×MIC AUR, 95.1% for PEITC combined with 4×MIC AUR, indicating that this combination showed strong biofilm inhibitory effects against RN450 at 24 h compared to AUR monotherapy (**Figures 6B, C**; P < 0.01). As with our results, such inhibition was absent in PEITC alone. These findings strongly supported the high efficacy of AUR and PEITC combination by interfering with biofilm formation.

### AUR-Induced Protection Against Skin Infections Is Enhanced in the Presence of PEITC

**Figure 7:**
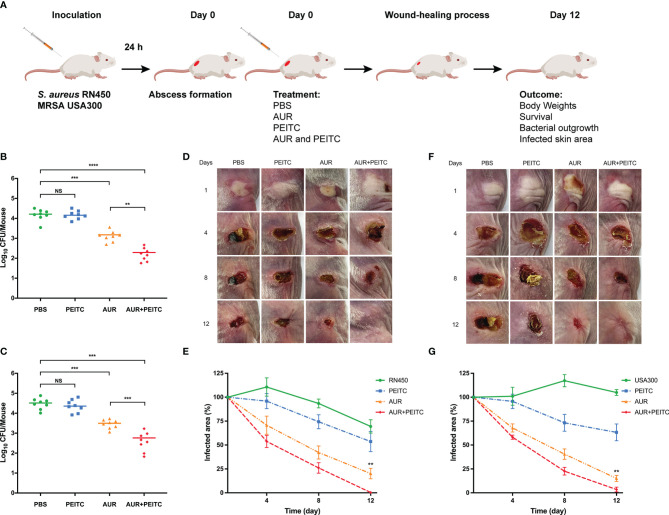
Effect of AUR-PEITC combination on skin infections caused by *S. aureus*. **(A)** Schematic diagram of skin infection and treatment process. **(B, C)** Bacterial burdens in treatment with PBS, AUR, PEITC or AUR-PEITC combination at 8 days after cutaneous challenge with **(B)** *S. aureus* RN450 and **(C)** MRSA USA300. **(D, F)** Digital photographs showing changes in the infected skin of mice at 0, 4, 8, 12 days after exposure to different treatments. **(E, G)** Quantification of the area of the infected skin of mice from each treatment group over time. **(D, E)** *S. aureus* RN450; **(F, G)** MRSA USA300. **(B, C, E, G)** Data are from three independent experiments. The group size was 8 mice. **(D, F)** One experiment is representative of three independent experiments. **P < 0.01, ***P < 0.001, ****P < 0.0001. Control, untreated control; NS, no significance; PBS, treated control. AUR, auranofin; PEITC, phenethyl isothiocyanate.

To definitively assess the antibacterial effects of AUR and PEITC *in vivo*, mouse models of subcutaneous abscess induced by *S. aureus* RN450 and MRSA USA300 were established (**Figure 7A**). USA300 is of particular concern given that this isolate has been linked to the majority of SSTIs in the USA (Stryjewski and Chambers, 2008). Successfully infected mice were divided into four groups and treated with PBS (control), AUR, PEITC, or both drugs together. As shown in **Figure 7**, we evaluated bacterial loads in the abscessed skin from each treatment group on the eighth day of treatment. Consistent with our *in vitro* results, AUR monotherapy showed antimicrobial activity against the infected bacteria but there was a sharp reduction in the burden of RN450 in response to AUR and PEITC together (**Figure 7B**). Similarly, a significant reduction in bacterial load was found in USA300-induced skin infections (**Figure 7C**).

Following treatment and lasted for 12 days, body weight (**Figure S5**) and survival rates (data not shown) were recorded every other day, but no significant differences were shown between the treatment groups for RN450 and USA300 (P > 0.05). To further verify the influence of combination therapy on subcutaneous abscess, the size of the infected skin area was measured after *S. aureus* challenge. As expected, the wound area of mice treated with the AUR and PEITC combination became significantly smaller, the skin healed well, and no obvious scars or ulceration were seen within 12 days as compared to AUR monotherapy against RN450 (**Figures 7D–F**; P < 0.01). AUR and PEITC treatment also resulted in a significantly smaller abscess area than AUR alone against USA300, with wounds that healed faster, and skin that grew back at an accelerated pace, leading to eventual loss of the abscess (**Figures 7E–G**; P < 0.01). These important observations reveal that AUR monotherapy is often inadequate when *S. aureus* forms high-density infections. AUR and PEITC together significantly improved treatment efficacy.

## Discussion

*S. aureus* is one of the most persistent bacterial pathogens with serious diseases, ranging from minor to life-threatening infections (Nowicka and Grywalska, 2019). *S. aureus*-induced SSTIs are common among patients seeking inpatient and outpatient medical care (Dayan et al., 2016; Inagaki et al., 2019), and recurrent infections are reported in nearly half of cases (Montgomery et al., 2015). While the incidence of MRSA in hospitalized patients has declined, the emergence of community-acquired MRSA poses a new challenge (Calfee, 2017). This has necessitated the development of effective antimicrobial agents against *S. aureus*. Combination treatment is a promising way to improve efficacy and reduce side effects and cytotoxicity. In this study, we evaluated the effectiveness of using AUR and PEITC to treat skin infections both *in vitro* and *in vivo*, and elucidated the mechanism of this treatment approach.

Previous studies found that AUR exhibits antimicrobial activity against Gram-positive bacteria (Aguinagalde et al., 2015; Tharmalingam et al., 2019). Here, we reported that AUR combined with PEITC increased the antimicrobial susceptibility of *S. aureus* pathogens to AUR by 8-fold. Remarkably, combination therapy resulted in the synergistic killing of antibiotic susceptible *S. aureus* and MRSA, including clinically isolated MRSA. These data strongly suggest that PEITC can enhance AUR-mediated protection against MRSA strains *in vitro*. Likewise, AUR in combination with PEITC also displayed a significant synergistic effect for VRSA Mu50, suggesting that combination treatment has antimicrobial activity against both susceptible and resistant *S. aureus* phenotypes. Our findings may also imply that the mechanism of action of AUR and PEITC is unique from any used antibiotics. Further research is needed to clarify the mechanism(s) of action of this drug combination against *S. aureus* pathogens.

During prolonged incubation with high AUR concentrations, we showed the considerably increase in its MIC value for all tested *S. aureus* isolates, which may accelerate the emergence of AUR-resistant bacterial strains. Consistent with our previous study, this probably occurred because *trx*B mutation results in loss of the primary target of AUR (Chen et al., 2020). However, no noticeable alterations to AUR MICs were observed when alongside PEITC. Further studies are needed to determine whether the presence of PEITC recover the inhibitory effect of AUR on the TrxR. This observation raises the possibility that PEITC could potentially regulate the redox balance through the expression of *trx*B in *S. aureus*. Notably, there is no results regarding its effect on Trx gene (*trx*A). Thus, as a better choice for treatment than AUR alone, the AUR and PEITC combination could aggravate destruction of the Trx-TrxR system, and cause subsequent bacterial death. However, the underlying mechanism of the synergistic antimicrobial activity of AUR combined with PEITC remains uncertain.

AUR can compromise the thiol-redox system to increase ROS levels (Harbut et al., 2015; Zhu et al., 2020). Consistent with these results, an obvious increase in ROS production was observed after treatment with AUR alone. However, in the absence of additional oxidative stress, bacteria can still maintain redox homeostasis by utilizing available cellular antioxidants, which may explain the bacteria regrowth observed after AUR monotherapy at 24 h. Meanwhile, ROS are also recognized as critical for cell death by PEITC (Lv et al., 2020b). However, we did not observe changes in ROS generation with PEITC alone, which may be due to the fact that lower concentrations were used in this study. It was recently proposed that fenton-mediated hydroxyl radical formation is a common mechanism in bacterial cell death; however, the contribution of ROS to bacterial killing remains controversial (Van Acker and Coenye., 2017; Keren et al., 2013). Based on the data, we obtained in the current study, together with data published by other groups, we proposed that synergistic antimicrobial activity induced by AUR and PEITC was linked to ROS accumulation. Not surprisingly, AUR and PEITC greatly enhanced intracellular ROS levels. This also shows that in the presence of additional oxidative stress, such as ROS production induced by PEITC, bacterial cells can no longer maintain their redox balance, allowing oxidative damage to occur. Results from this study showed that DMSO is a scavenger of ROS, reducing the bacterial killing caused by AUR and PEITC, and suggesting that ROS plays a critical role in the synergistic killing.

Interestingly, after the addition of DMSO, combination treatment was still more effective than AUR monotherapy. This suggests that mechanisms aside from ROS regulation are involved. Indeed, AUR and PEITC gave rise to observable structural alterations ranging from ruffling of the cell walls to lysis, large-range membrane rupture, and the presence of cell debris when viewed using SEM, which indicates that this drug combination can potentially impair cell integrity or morphology with consequent positive effects on antimicrobial activity. Moreover, biofilms formation is related to bacterial drug resistance, and has been reported as the cause of many chronic bacterial infections, especially *S. aureus*. It could form recalcitrant biofilms to evade antibiotics and help persister cells growth, which demonstrates that entire biofilm structures must be eradicated for successful elimination of *S. aureus* pathogens (Yan and Bassler, 2019; Dong et al., 2019). We made the striking observation that effect of the AUR and PEITC combination on biofilm formation. As we expected, this combination showed higher biofilm inhibition rates when compared to AUR monotherapy. Overall, our data revealed that an increase in ROS production, more cellular structural alterations, and strong biofilm inhibition contributed to the synergistic activity of AUR and PEITC.

*S. aureus*, in particular MRSA, is a leading cause of skin infections. Chaney et al. reported that *S. aureus* blocks keratinocyte proliferation and migration, delaying wound closure (Chaney et al., 2017). As a result of the potent antibacterial activity exhibited by AUR and PEITC *in vitro*, we decided to further investigate the *in vivo* efficacy of this combination in treating MRSA skin infections. Findings confirmed that AUR and PEITC can significantly reduce the number of bacteria colonies under phase callus, accelerate cutaneous wound healing, and promote resistance to infection and healing efficacy compared to AUR monotherapy. However, PEITC alone had no impact on the healing process. This suggests that the AUR and PEITC drug combination can improve skin wound healing by eliminating *S. aureus*, including MRSA.

The combination of AUR and PEITC induced antimicrobial activity against *S. aureus* isolates was confirmed by *in vitro* and *in vivo* data. This synergistic effect is mainly achieved by promoting intracellular ROS accumulation, exacerbating the destruction of bacterial cell structures, and inhibiting biofilm formation. Remarkably, these findings highlighted that AUR-PEITC combination could serve as a potential therapeutic scheme for *S. aureus* skin infection. Future investigates other diseases caused by *S. aureus* (including endocarditis, bacteremia, and osteomyelitis) are of great importance to prevent clinical progression as well as the spread of the infection, especially considering the crucial role of this drug combination in treating MRSA skin infection.

Taken together, we have demonstrated that AUR and PEITC act synergistically to kill *S. aureus* strains, and slow down the development of AUR resistance *via* reducing TrxR gene expression. Importantly, the *in vitro* antimicrobial potency of AUR and PEITC could be well-translated *in vivo*. Our work clearly elucidates the antimicrobial action of the AUR-PEITC combination and opens a path for the treatment of infections caused by *S. aureus* strains, especially those that are drug resistant.

## Conclusion

The AUR-PEITC combination exhibits synergistic antimicrobial activity against *S. aureus* pathogens *in vitro* and *in vivo* by enhancing ROS generation, aggravating cellular structural alterations, and blocking biofilm generation while preventing the occurrence of AUR resistance. Given that AUR and PEITC are currently available, using this combination would be more efficient than developing new antimicrobial drugs. This work has the potential to make a significant contribution to infectious disease management.

## Data Availability Statement

The original contributions presented in the study are included in the article/**Supplementary Material**. Further inquiries can be directed to the corresponding authors.

## Ethics Statement

The animal study was reviewed and approved by the Animal Experimentation Ethics Committee of Anhui Medical University.

## Author Contributions

HC and JBL conceived the idea, designed the study. HC, NY, and YY performed the experiments, analyzed the data and wrote the manuscript. HZ, YZ, MX, and YL provided technical support. HC, JBL, JJL, and LY revised the manuscript. All authors contributed to the article and approved the submitted version.

## Funding

The work was supported by the National Natural Science Foundation of China (No. 81973983), Collaborative Tackling and Public Health Collaborative Innovation Project in Anhui Province (No. GXXT-2020-018), the Joint Construction Project of Clinical Medicine University and Hospital (No. 2021lcxk006), Natural Science Research Project of Universities in Anhui Province (No. KJ2020A0176), and Postgraduate Research Project of Universities in Anhui Province (No.YJS20210267).

## Conflict of Interest

The authors declare that the research was conducted in the absence of any commercial or financial relationships that could be construed as a potential conflict of interest.

## Publisher’s Note

All claims expressed in this article are solely those of the authors and do not necessarily represent those of their affiliated organizations, or those of the publisher, the editors and the reviewers. Any product that may be evaluated in this article, or claim that may be made by its manufacturer, is not guaranteed or endorsed by the publisher.
