# Supplementary material for: Synergistic Microbicidal Effect of AUR and PEITC Against Staphylococcus aureus Skin Infection
Source: Front Cell Infect Microbiol. 2022 Jun 14;12:927289. doi: 10.3389/fcimb.2022.927289 (PMC9237442; doi:10.3389/fcimb.2022.927289)
Supplement: Supplementary file 1 [file DataSheet_1.pdf]

Supplementary Material

1 Supplementary Figures and Tables

1.1 Supplementary Figures

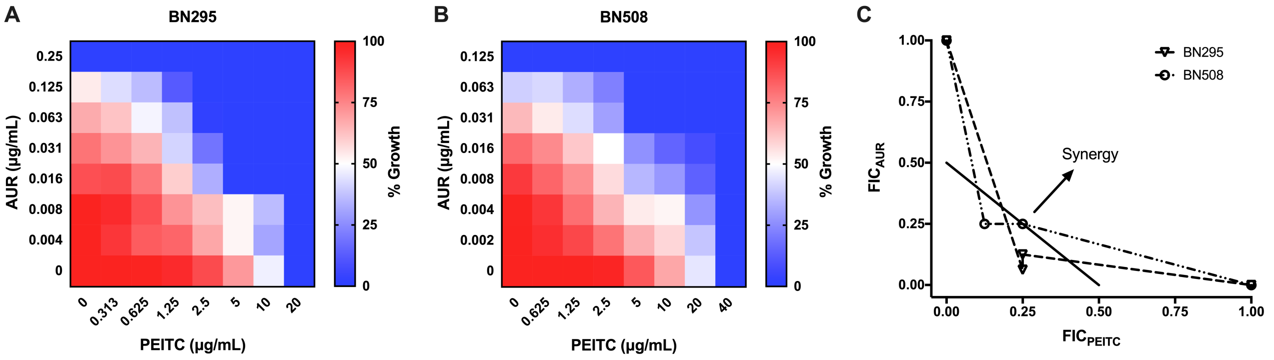

Supplementary Figure 1. Synergistic activity of AUR combined with PEITC for MRSA clinical strains. Representative heat plots of checkerboard assays for the combination of AUR and PEITC against **(A)** MRSA BN295 and **(B)** MRSA BN508. **(C)** Isobolograms of the combination of AUR and PEITC against MRSA strains. The black full line indicates ideal isobole, where drugs act additively and independently. Data points below this line indicate synergism.

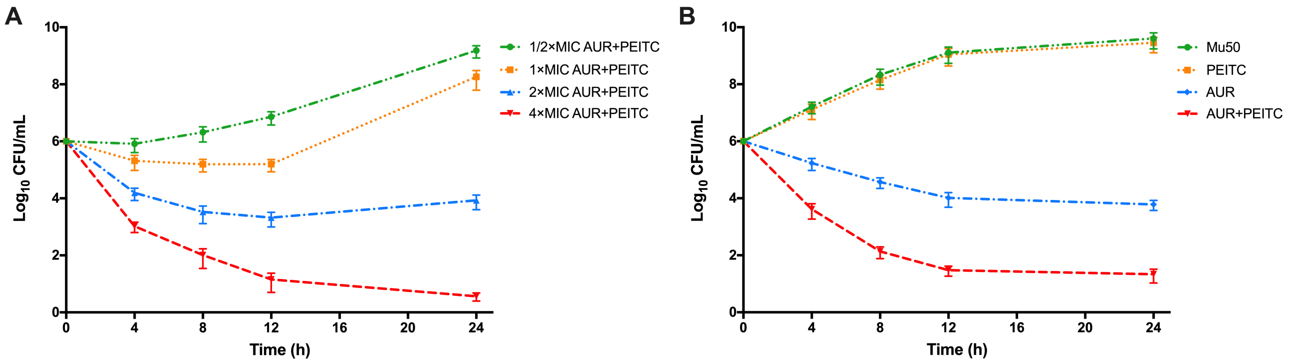

Supplementary Figure 2. Time-kill assays of *S. aureus* by AUR in combination with PEITC. **(A)** Exponentially growing cultures were treated with various concentrations of AUR (1/2× MIC, 1× MIC, 2× MIC and 4× MIC) in combination with PEITC (1× MIC) for *S. aureus* RN450; **(B)** treated with 4× MIC AUR or 1× MIC PEITC against VRSA Mu50. Data are from at least three independent experiments; error bars indicate the standard errors of the means.

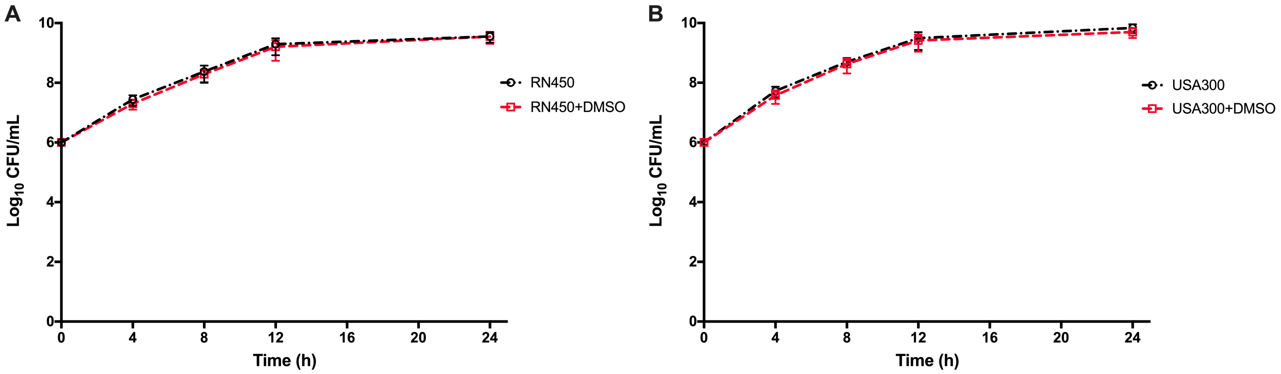

Supplementary Figure 3. The effect of DMSO on *S. aureus* RN450 growth kinetics. The addition of DMSO (5 %) alone did not alter bacterial growth *in vitro*. Results are representative of three independent experiments; error bars represent the standard errors of the means. P values (compared to untreated) are > 0.05.

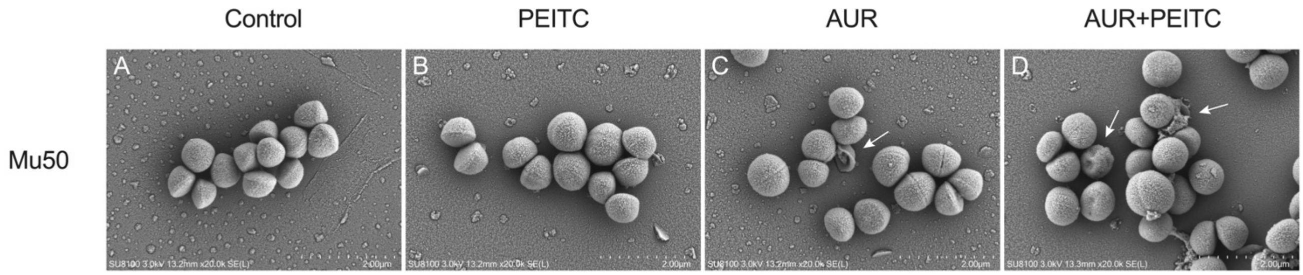

Supplementary Figure 4. Cellular morphology of VRSA Mu50. SEM images of VRSA Mu50 after treatment with **(B)** 4× MIC AUR alone, **(C)** 1× MIC PEITC alone or **(D)** combination. **(A)** represent the control condition.

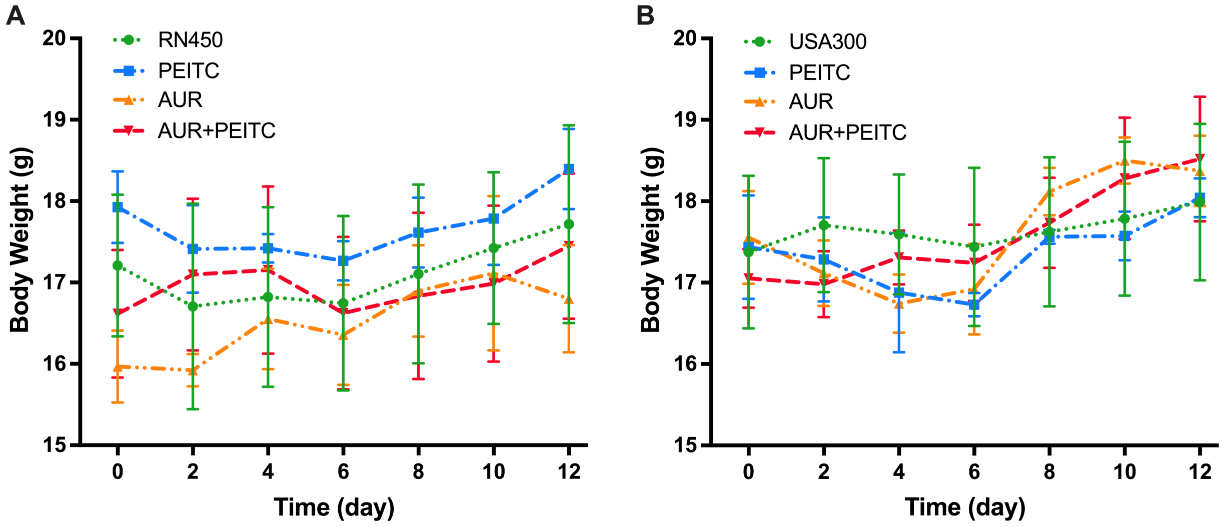

Supplementary Figure 5. Mouse survival curves of AUR-PEITC combination. AUR combined with PEITC did not impact on survival rates of skin infections caused by **(A)** *S. aureus* RN450 and **(B)** MRSA USA300.

1.2 Supplementary Tables

Supplementary Table 1. Primers of gene expression by real-time RT–PCR.

Gene	Forward primer (5' to 3')	Reverse primer (5' to 3')
*trx*A	TCCATGTAAAATGATCGCTCCG	TTTATCAACTGGTTGACCGTCTTT
*trx*B	TCGTTCACCGTCGTGATGAG	ACCATCAGCCTCGTGTGTTT
16S-qPCR[a]	GTTTGTAACACCCGAAGCCG	ACCTTCCGATACGGCTACCT

[a]16S ribosomal RNA
